# Defining the overlap between sporadic and attenuated familial adenoma risk

**DOI:** 10.1186/1897-4287-9-S1-P37

**Published:** 2011-03-10

**Authors:** Thérèse M Tuohy, Deborah W Neklason, Kenneth M Boucher, Richard G Pimentel, Kerry G Rowe, Geraldine P Mineau, Randall W Burt

**Affiliations:** 1 High Risk Cancer Clinics, Huntsman Cancer Institute, University of Utah, Salt Lake City, UT, USA; 2 Dept. Oncological Sciences, Huntsman Cancer Institute, University of Utah, Salt Lake City, UT, USA; 3 Dept. of Oncology, Intermountain Healthcare, Salt Lake City, UT, USA; 4 Dept. of Medicine, Huntsman Cancer Institute, University of Utah, Salt Lake City, UT, USA

## Background

Mutations in the *APC* gene lead to Familial adenomatous polyposis (FAP) and an attenuated form of this condition (AFAP). Based on previous work with mutation-verified patients from a large AFAP kindred, we showed that AFAP patients may be under-diagnosed, in the absence of a genetic diagnosis. For example, for patients between 30 and 79 years of age, 28% had fewer than 10 adenomas; 22% had fewer than 6 adenomas, and 7% had no polyps. Therefore, depending on the number of adenomas considered more consistent with sporadic, rather than a familial predisposition, and the availability or lack of known family history, up to ~25% of mutation-carrying AFAP patients may be missed in clinical practice. We sought to address this challenge by profiling mutation-independent adenoma formation among the mutation-negative carriers in this kindred.

## Methods

Careful endoscopic evaluation of the AFAP cases has been previously reported, and these data were used for the mutation-positive individuals. The Utah Population Database (UPDB) includes Utah genealogies linked with medical records serving 85% of the Utah population. The database was queried for all descendents of known mutation-negative branches of this large kindred who had undergone colonoscopy procedures between 1995 and 2009. The queries collected data on age, gender, polyp type, pathology and cancer diagnoses, and were verified by manual review of randomly selected cases. Multiple logistic regression allowed us to characterize the relative risk of a mutation, based on the adenoma profile of mutation carriers *vs.* non-carriers and additional covariates.

## Results

A total of 135 colonoscopy records were collected and reviewed for 65 individuals who subsequently tested negative for the family mutation, at a median age of 51 (standard deviation of 16 years) and average pathology-confirmed adenoma count of 0.25 (mode = 0) at first colonoscopy. For patients between 30 and 79 years of age, 5% had 2 or more adenomas; 17% had a single adenoma, and 79% had no adenomas. Additional comparable data were available on 130 individuals from negative branches of the kindred, who underwent colonoscopy between 1995 and 2009.

## Conclusions

The combination of a long-running study with archived records and a large, electronically searchable medical database with a linked genealogical resource offers a powerful platform for the development of hypothesis-driven research. This resource may be used to address both clinical and research questions whose answers may be used to guide clinical management.

## Disclosure and funding

There are no financial interests to disclose. This research was funded by NIH grants P01CA073992 (RWB); the Utah Population Database and the Huntsman Cancer Foundation.

